# Family Caregiving Subtypes in the Caregiving Transitions Study: A Latent Class Analysis

**DOI:** 10.1093/geroni/igab046.2923

**Published:** 2021-12-17

**Authors:** John Bentley, Carly Lupton-Smith, David Roth

**Affiliations:** 1 University of Mississippi, University of Mississippi, Mississippi, United States; 2 Johns Hopkins University, Johns Hopkins University, Maryland, United States; 3 Johns Hopkins University, Baltimore, Maryland, United States

## Abstract

Family caregiving requires activities and experiences that have negative and positive features, producing stress but also providing benefits. The Caregiving Transitions Study (CTS) enrolled 283 caregivers from a national epidemiologic study, of which 32 were caregivers prior to enrollment in the parent study, and 251 became caregivers while participating in the parent study. Telephone interviewers were conducted after caregivers provided care for a minimum of 1.6 years (mean=7.7 years). Latent class analysis (LCA) was used to detect unobserved groups of caregivers. Number of problems (i.e., ADL, IADL, communication, emotional, disruptive behavior), average burden per problem, depressive symptoms, perceived stress, purpose in life, positive aspects of caregiving, hours of care, and duration providing care were used as indicators. Classes were subsequently compared on several external variables, including demographics, quality of life, leisure activities, and caregiving strain. The best-fitting model consisted of three classes (4.6% long-term, 27.6% high-distress, and 67.8% moderate-distress). Classes were similar with respect to sex, age, race, and primary caregiving status of the caregiver. Long-term caregivers had much longer caregiving durations and commonly provided care to a child. The high-distress class was noteworthy in terms of greater number of experienced patient problems; greater likelihood of caring for a person with dementia; higher levels of caregiving strain, depressive symptoms, perceived stress, and perceived burden; and lower levels of quality of life, purpose in life, positive aspects of caregiving, and leisure activities. These findings suggest that caregivers can be classified into distinct subtypes, with one subtype characterized as experiencing high distress.

